# Chemerin plasma levels are increased in COVID-19 patients and are an independent risk factor of mortality

**DOI:** 10.3389/fimmu.2022.941663

**Published:** 2022-08-12

**Authors:** Philomène Lavis, Sofia Morra, Carmen Orte Cano, Nurhan Albayrak, Véronique Corbière, Véronique Olislagers, Nicolas Dauby, Véronique Del Marmol, Arnaud Marchant, Christine Decaestecker, Françoise Mascart, Nathalie De Vos, Philippe Van de Borne, Isabelle Salmon, Myriam Remmelink, Marc Parmentier, Alessandra Kupper Cardozo, Benjamin Bondue

**Affiliations:** ^1^ Department of Pathology, Erasme Hospital, Université libre de Bruxelles, Brussels, Belgium; ^2^ I.R.I.B.H.M., Université libre de Bruxelles, Brussels, Belgium; ^3^ Department of Cardiology, Erasme Hospital, Université libre de Bruxelles, Brussels, Belgium; ^4^ Department of Dermatology, Erasme Hospital, Université libre de Bruxelles, Brussels, Belgium; ^5^ Laboratory of Vaccinology and Mucosal Immunity, Université libre de Bruxelles, Brussels, Belgium; ^6^ Institute for Medical Immunology, Université libre de Bruxelles, Brussels, Belgium; ^7^ Department of Infectious Diseases, C.H.U. Saint-Pierre, Brussels, Belgium; ^8^ DIAPath, Center for Microscopy and Molecular Imaging, Université libre de Bruxelles, Gosselies, Belgium; ^9^ Laboratory of Image Synthesis and Analysis, Université libre de Bruxelles, Brussels, Belgium; ^10^ Department of Clinical Chemistry, LHUB-ULB, Université libre de Bruxelles, Brussels, Belgium; ^11^ Institute for Translational Research in Cardiovascular and Respiratory Sciences, Université libre de Bruxelles, Brussels, Belgium; ^12^ Centre Universitaire inter Régional d’expertise en Anatomie Pathologique Hospitalière, Jumet, Belgium; ^13^ Inflammation and Cell Death Signalling group, Experimental Gastroenterology Laboratory and Endotools, Université libre de Bruxelles, Brussels, Belgium; ^14^ Department of Pneumology, Erasme Hospital, Université libre de Bruxelles, Brussels, Belgium

**Keywords:** COVID-19, chemerin, ChemR23, CMKLR1, survival, ARDS, viral pneumonia, prediction

## Abstract

**Background:**

Chemerin is an extracellular protein with chemotactic activities and its expression is increased in various diseases such as metabolic syndrome and inflammatory conditions. Its role in lung pathology has not yet been extensively studied but both known pro- and anti-inflammatory properties have been observed. The aim of our study was to evaluate the involvement of the chemerin/ChemR23 system in the physiopathology of COVID-19 with a particular focus on its prognostic value.

**Methods:**

Blood samples from confirmed COVID-19 patients were collected at day 1, 5 and 14 from admission to Erasme Hospital (Brussels – Belgium). Chemerin concentrations and inflammatory biomarkers were analyzed in the plasma. Blood cells subtypes and their expression of ChemR23 were determined by flow cytometry. The expression of chemerin and ChemR23 was evaluated on lung tissue from autopsied COVID-19 patients by immunohistochemistry (IHC).

**Results:**

21 healthy controls (HC) and 88 COVID-19 patients, including 40 in intensive care unit (ICU) were included. Plasma chemerin concentration were significantly higher in ICU patients than in HC at all time-points analyzed (p<0.0001). Moreover, they were higher in deceased patients compared to survivors (p<0.05). Logistic univariate regression and multivariate analysis demonstrated that chemerin level at day 14 of admission was an independent risk factor for death. Accordingly, chemerin levels correlated with inflammatory biomarkers such as C-reactive protein and tumor necrosis factor α. Finally, IHC analysis revealed a strong expression of ChemR23 on smooth muscle cells and chemerin on myofibroblasts in advanced acute respiratory distress syndrome (ARDS).

**Discussion:**

Increased plasma chemerin levels are a marker of severity and may predict death of COVID-19 patients. However, multicentric studies are needed, before chemerin can be considered as a biomarker of severity and death used in daily clinical practice. Further studies are also necessary to identify the precise mechanisms of the chemerin/ChemR23 system in ARDS secondary to viral pneumonia.

## Introduction

Chemerin is an extracellular protein first described in the early 2000’s (1). It is secreted as an inactive precursor (prochemerin), by many cell types including adipocytes, hepatocytes, fibroblasts and epithelial cells (2–4). The active form of chemerin is generated by the removal of six or seven amino-acids from its carboxy-terminus by serine proteases, such as cathepsin G and elastase that are produced by neutrophils (5). Chemerin binds to three receptors: chemokine like receptor 1 (CMKLR1 also named ChemR23), G protein-coupled receptor 1 (GPR1) and chemokine (C-C motif) receptor-like 2 (CCRL2) (6). ChemR23 mediates the main effects of chemerin, while the binding of chemerin to GPR1 and CCRL2 results respectively in weak signaling or no detectable cell activation (6). ChemR23 is expressed in different cell types such as macrophages, immature myeloid dendritic cells (mDC), immature plasmacytoid dendritic cells (pDC), natural killer (NK) cells, endothelial cells (EC), pericytes, adipocytes and smooth muscle cells (2, 7–9).

Circulating chemerin levels are increased in various pathologies notably in metabolic syndrome, inflammatory conditions such as rheumatoid arthritis, inflammatory bowel diseases and cancers (10–13). The chemerin/ChemR23 system has both pro- and anti-inflammatory properties depending probably on the tissue where it is activated and the stimulus. Chemerin was first described as a major chemoattractant agent, particularly for pDCs, involved in antiviral responses through the secretion of type I interferons (IFN I) (1, 4). In a murine model of viral pneumonia, we observed that ChemR23 knock-out (KO) mice exhibited a lower recruitment of pDCs and a delayed viral clearance but also an excess mortality and increased neutrophil infiltration (14), suggesting an anti-inflammatory role for chemerin. This was further confirmed in a mouse model of acute lung injury induced by the intratracheal administration of bacterial lipopolysaccharide (LPS). In this model, we showed that treatment with recombinant chemerin led to a lower recruitment of neutrophils in lungs, decreased the severity of histological lesions and the release of pro-inflammatory cytokines (4). On the other hand, the pro-inflammatory role of this system was also highlighted in a mouse model of lung inflammation secondary to cigarette exposition. In this case, ChemR23 KO mice presented lower innate and adaptive immune responses compared to wild-type mice (15). Altogether, these experiments revealed an important role for the chemerin/ChemR23 system in lung inflammation, which is not yet completely elucidated.

The COVID-19 pandemic is caused by a coronavirus named SARS-CoV-2 (16). Coronaviruses are well-known to be responsible for acute respiratory distress syndrome (ARDS) and SARS-CoV-2 induces a high hospitalization (20%) and mortality rate (1%) in non-vaccinated individuals (17). Symptomatology is, however, extremely variable from the absence of symptoms to the development of an ARDS with multiorgan failure (18, 19). Lesions in these patients do not seem to be related to viral load, but to a hyperinflammatory state characterized by an excessive secretion of pro-inflammatory cytokines such as interleukin 6 (IL-6) associated with a decrease of antiviral cytokines like IFN I (20). Risk factors for the development of a severe COVID-19 outcome have been identified such as age, the presence of a metabolic syndrome, chronic lung, liver or kidney diseases and immunosuppression (21).

Given that chemerin is elevated in most of the pathologies associated with a higher risk of developing severe COVID-19 and seems to be associated with lung inflammation, in the present study, we evaluated the chemerin/ChemR23 system in COVID-19 patients and correlated it with disease severity.

## Materials and methods

### Study design

This prospective observational study was conducted on adult patients (from 18 to 70 years) with a confirmed COVID-19 infection (positive polymerase chain reaction (PCR) test performed on a nasopharyngeal swab), admitted to Erasme Hospital (Tertiary care center, Brussels, Belgium). These patients were further classified into three groups: non-hospitalized (NH), hospitalized in a conventional care unit (H) and hospitalized in intensive care unit (ICU). The study was approved by the local Ethical Committee (P2020/238 for blood biobanking, P2020/232 for bronchoalveolar lavage biobanking and immunohistochemistry analysis).

#### Collection of blood and bronchoalveolar lavage samples and clinical data

Blood samples from confirmed COVID-19 patients were collected from April to December 2020 and stored at the Biobank BB190012 from the Laboratory of Vaccinology and Immunology at the Université libre de Bruxelles (ULB), Brussels, Belgium. Three different time points were analyzed: day 1 (D1), day 5 (D5) and day 14 (D14) from admission. As controls, blood samples from health care workers participating to a seroprevalence study (22) in another hospital located in the Brussels Capital Region were used (asymptomatic with negative PCR and serological tests (IgG and IgA)). Within two hours after collection, plasma from EDTA tube was extracted and stored at -20°C and whole blood from another tube was mixed with Cytodelics Stabiliser (Cytodelics AB, Stockholm, Sweden) and stored at -80°C, as previously described (23). Covid and control samples were prepared and stored identically.

Clinical data were obtained from patient’s medical records with a focus on the following characteristics: age, gender, body mass index (BMI), smoking, comorbidities associated with a higher risk to develop a severe COVID-19 (hypertension, diabetes, chronic obstructive pulmonary disease (COPD), chronic kidney disease and immunosuppression), peripheral oxygen saturation (SpO2) at admission, the need of ventilation, the development of an ARDS, the duration of hospitalization and outcome. Of note, ARDS was defined according to Berlin criteria as the development of breathing difficulties with hypoxemia (arterial partial pressure of oxygen to fraction of inspired oxygen (PaO2/FIO2) <300 mmHg) associated with bilateral opacities on chest imaging in the absence of cardiac failure (24). The results of routine blood analysis were collected from the medical files including C-reactive protein (CRP), white blood cell count (WBC), polymorphonuclear cells (PMN), lymphocytes, platelets, ferritin, lactate dehydrogenase (LDH), creatinine kinase (CK), alanine transaminase (ALT), aspartate transaminase (AST), gamma-glutamyl transferase (GGT), creatinine, total cholesterol and triglycerides.

Another cohort of patients was used for bronchoalveolar lavages (BAL) collection. In this cohort, BALs were performed to confirm a SARS-CoV-2 infection or exclude opportunistic superinfections (immunocompromised patients). BAL were collected from April 2020 to November 2020. They were performed, using a disposable video-bronchoscope (Ambu^®^ aScopeTM, Ballerup, Denmark) as described by Taton et al. (25). Samples were centrifuged (1400 rpm – 10 minutes), then supernatants were stored at -80°C and cell pellets stabilized with Cytodelics stabilizer and then stored at -80°C (Biobank ULB-COVID-19, BB200022). Only confirmed COVID-19 patients from this cohort were selected for further analysis. Control BAL samples were collected from patients with a pulmonary nodule or from non-infected lung transplant patients (performed as part of their routine follow up). Neutrophils, lymphocytes and macrophages counts on BAL were obtained from the medical files.

#### Analysis of peripheral blood and BAL samples

Plasma and BAL chemerin levels were assessed by enzyme-linked immunosorbent assay (ELISA) (R&D systems, Minneapolis, MN). Samples were analyzed in duplicates according to the manufacturer’s instructions. Plasma levels of the Krebs Von den Lungen protein (KL-6) were detected using a sandwich ELISA assay on Lumipulse G600II and G1200 (Fujirebio, Japan). The concentrations of IFN-α, IFN-γ, IL-1β, IL-6, IL-7, IL-8, IL-10, IL-17α, monocyte chemoattractant protein-1 (MCP-1, CCL2), monokine induced by interferon-γ (MIG, CXCL9), macrophage inflammatory protein-1β (MIP-1β, CCL4) and tumor necrosis factor α (TNFα) were measured in plasma by multiparameter-based immunoassays (Milliplex Human Cytokine/chemokine/Growth factor panel A magnetic bead panel kit-Merck, Belgium) according to the manufacturer’s instructions. Results were analyzed with a Bio-Plex 200^®^ Multiplex reader, Bio-Plex ManagerTM Manager 4.1 Software (BIO-RAD laboratories, Nazareth Eke, Belgium).

Whole blood cells conserved into the Cytodelics Stabilizer were prepared for flow cytometry analysis according to the manufacturer’s instructions. After complete lysis of red blood cells, the immune cells were stained with antibodies from Thermofisher (Waltham, MA, USA): CD45 (EF450), CD3 (EF506), CD123 (SB600), CD11c (SB645), HLA-DR (SB702), CD16 (SB780), CD14 (FITC), CD141 (PE-Cy7), CD56 (APC-EF780) and ChemR23 (APC). Flow cytometry analysis were performed on an LSR Fortessa instrument (BD Biosciences, Franklin Lakes, NJ, USA) and analyzed using FlowJo software (BD Biosciences, Franklin Lakes, NJ, USA) to identify different immune cell populations and their expression of ChemR23. The gating strategy was previously described by Albayrak et al. (23).

#### Lung tissue sample collection and immunohistochemistry

Formalin-fixed paraffin-embedded (FFPE) lung tissues from PCR positive COVID-19 patients stored in the Biobank ULB-COVID-19 (Department of Pathology, Erasme Hospital, BB200022) were used for immunohistochemical (IHC) staining. Autopsies were performed after 72 hours to prevent infection of the medical staff (26). For this analysis, areas presenting typical lesions of diffuse alveolar damage (DAD) early or late and samples with pneumonia or bronchopneumonia were selected. Control tissues were obtained from autopsied patients deceased for more than 48 hours without major lung lesion and patients deceased of an ARDS from another origin.

IHC staining was performed using an automated immunostainer (Dako Omnis, Agilent Technologies, Santa Clara, CA, USA). Thus, 4µm slides were incubated respectively with anti-ChemR23 (Santa Cruz Biotechnology, sc-398769, dilution 1:300) and anti-chemerin (Santa Cruz Biotechnology, sc-373797, dilution 1:100) antibodies and counterstained with hematoxylin. Then slides were scanned with a 40x magnification (Nanozoomer, Hamamatsu, Hamamatsu-City, Japan) before assessment by two pathologists.

### Statistical analysis

All data were tested for normality using the Shapiro-Wilk test and according to the distribution, parametric or non-parametric tests were applied. Differences between the groups were assessed by one-way ANOVA or Kruskal-Wallis test. Tukey or Dunn’s tests were used as *post-hoc* tests. When only two groups were compared, student t or Mann-Whitney tests were used. Data are reported as mean ± standard deviation (SD) or median with a confidence interval of 95% (CI 95%). Categorical variables are listed as numbers with percentage. Chi-square test was performed to compare more than two groups and Fisher’s exact test was used for comparison between 2 groups. For correlation analysis, non-parametric Spearman correlation was performed, and data are reported as Spearman r and p-value. To determine the association of different variables to fatal outcome, a univariate logistic regression test was performed and then a multivariable regression model was chosen from a stepwise selection based on the Akaike Information Criterion (AIC). The analyses were performed on Prism 6 or SAS 9.4 software packages and all significance levels were fixed at 0.05.

## Results

### Baseline characteristics of patients

In this study 88 patients with a positive nasopharyngeal throat swab for COVID-19 were included: 11 non-hospitalized (NH), 37 hospitalized in a conventional care unit (H) and 40 hospitalized in intensive care unit (ICU). Blood samples were collected at the admission D1, D5 and D14. Symptoms started on average 9 days before admission to the emergency department. Consequently, samples obtained at D1, D5 and D14 correspond to day 9, 14 and day 23 after initial symptoms. 21 healthy subjects (HC) (asymptomatic with negative PCR and serological tests (IgG and IgA)) were used as controls.

Baseline characteristics of controls and patients are reported in **Table 1** and multiple comparisons between subgroups are reported in **Supplemental Table 1**. Sixty-seven patients were men and 42 were women. Patients were older in H and ICU groups (57.6 ± 14.8 and 60.5 ± 10.9, respectively, mean ± SD) as compared to HC and NH groups (38.6 ± 7.9 and 42.2 ± 10.9 respectively; mean ± SD, p<0.0001). No significant differences were found for BMI among COVID-19 patients (average BMI for NH, H and ICU groups: 28.6 kg/m² (27.2-30.45), median (95% CI)) as well as for tobacco use (18.1% of the patients).

**Table 1:** Baseline characteristics and comorbidities.

Baseline characteristics and comorbidities	Healthy controls (n=21)	Non-hospitalized COVID-19 patients (n=11)	Hospitalized non-ICU COVID-19 patients (n=37)	ICU COVID-19 patients (n=40)	p-value
Age (years)°	38.6 ± 7.9	42.2 ± 10.9	57.6 ± 14.8	60.5 ± 10.9	**<0.001**
Gender (M/F)	8/3	8/3	24/13	31/9	**<0.001**
BMI (kg/m²)^$^	–	28.4 (22.4-33.9)	27.7 (26.0-30.8)	29.5 (27.6-33.1)	0.117
Smoking n (%)	–	2 (18)	4 (12)	8 (25)	0.379
Hypertension n (%)	0 (0)	2 (18)	19 (51)	24 (60)	**<0.001**
Diabetes n (%)	0 (0)	1 (9)	10 (27)	23 (57.5)	**<0.0001**
Immunosuppression n (%)	0 (0)	0 (0)	5 (13.5)	5 (12.5)	0.088
COPD n (%)	0 (0)	0 (0)	4 (11)	2 (5)	0.303
CKD n (%)	0 (0)	0 (0)	6 (16)	2 (5)	0.327
Dexamethasone n (%)	–	0 (0)	20 (54)	35 (87.5)	**<0.001**

°Parametric data presented as mean ± Standard deviation (SD). ^$^Non-parametric data presented as median with confidence interval of 95%. BMI, body mass index; CKD, chronic kidney disease; COPD, chronic obstructive pulmonary disease. Statistics analysis was performed using one-way ANOVA or Kruskal-Wallis test, according with the distribution of the data, for non-categorical variables. Chi-square test was applied for categorical variables. Values in bold indicate statistical significance.

Routine biological analyses of the COVID-19 patients are reported in **Table 2** and multiple comparisons between subgroups are reported in **Supplemental Table 2**. At admission and without oxygen support, the median peripheral oxygen saturation (SpO2) decreased with severity of disease and therefore was the lowest in the ICU group (p<0.0001) (**Table 2**). Inflammation-related parameters such as CRP (p<0.0001), ferritin (p<0.01), LDH (p<0.0001) and D-dimer (p<0.001) were higher and significantly different in plasma from ICU patients compared to NH and H patients. Liver tests were slightly disturbed in ICU patients with a small elevation of the transaminases. On the other hand, no alteration of the renal function or the lipids tests was observed. ICU patients had higher and significantly different levels of circulating WBC (p<0.0001) with a predominance of PMN (p<0.0001) and lower levels of lymphocytes (p<0.001).

**Table 2:** Main biological parameters at admission.

	Non-hospitalized COVID-19 patients (n=11)	Hospitalized non-ICU COVID-19 patients (n=37)	ICU COVID-19 patients (n=40)	p-value
KL-6 D1 (U/mL)^$^	301 (154-422)	377.5 (254-547)	577 (422-760)	**0.004**
SpO2 (%)^$^	99 (97-100)	94 (92-95)	88 (80-92)	**<0.001**
Hemoglobin (g/dL)°	13.2 ± 2.8	12.8 ± 1.9	12.6 ± 2.2	0.156
WBC (10³/mm³)^$^	5.0 (3.5-9.1)	5.3 (4.8-6.8)	11.3 (8.9-13.1)	**<0.001**
PMN (10³/mm³)^$^	2.9 (1.7-5.8)	4.3 (3.1-5.1)	8.9 (7.5-11.2)	**<0.001**
Lymphocytes (10³/mm³)^$^	1.5 (1.1-2.3)	0.8 (0.7-1.0)	0.8 (0.6-0.8)	**<0.001**
Platelets (10³/mm³)^$^	194 (115-270)	190.5 (153-213)	256 (214-336)	**<0.001**
CRP (mg/L)^$^	11.5 (1.9-59)	69 (38-88)	135 (97-180)	**<0.001**
Ferritin (µg/L)^$^	–	604 (386-1261)	1113 (834-1670)	**0.008**
LDH (U/L)^$^	214 (153-335)	324.5 (278-370)	435 (366-520)	**<0.001**
ALT (U/L)^$^	23 [17-33]	27 [21-30]	46 [27-59] (39)	**0.014**
AST (U/L)^$^	24 (17-38)	34.5 (24-41)	40 (34-55)	**0.019**
GGT (U/L)^$^	50.50 (48-53)	50 (33-67)	70 (43-105)	0.115
Total bilirubin (mg/dL)^$^	0.6 (0.2-0.9)	0.4 (0.4-0.5)	0.6 (0.4-0.7)	0.548
CK (U/L)^$^	188 (53-470)	91 (59-184)	129 (79-335)	0.342
D-dimer (ng/mL)^$^	–	543 (440-1041)	1182 (963-2528)	**<0.001**
Creatinine (mg/dL)^$^	0.9 (0.7-1.2)	0.8 (0.8-1.0)	0.9 (0.7-1.0)	0.793
Urea (mg/dL)^$^	24 (18.4-26.9)	33.4 (28.1-40.5)	41.7 (33.3-55.3	**0.002**
GFR (mg/dL/1,73m²)^$^	93 (69-118)	93 (82-100)	84.5 (69-99)	0.574
Cholesterol total (mg/dL)°	148.5 ± 55.72	158.7 ± 39.17	149 ± 39.7	0.705
Triglycerids (mg/dL)^$^	69 (10.3-162.7)	130 (117.4-166.2)	150.5 (131.7-237)	0.123

°Parametric data presented as mean ± Standard deviation (SD). ^$^Non-parametric data presented as median with confidence interval of 95%. ALT, alanine transaminase; AST, aspartate transaminase; CK, creatinin kinase; CRP, C-reactive protein; GFR, glomerular filtration rate (CKD-epi formula); GGT, gamma-glutamyl transferase; KL-6, Krebs Von den Lungen 6; LDH, lactate dehydrogenase; PMN, polymorphonuclear cell; SpO2, median peripheral oxygen saturation; WBC, white blood cell. Statistics analysis was performed using one-way ANOVA or Kruskal-Wallis test, according to the distribution of data. Values in bold indicate statistical significance.

**Figure 1:**
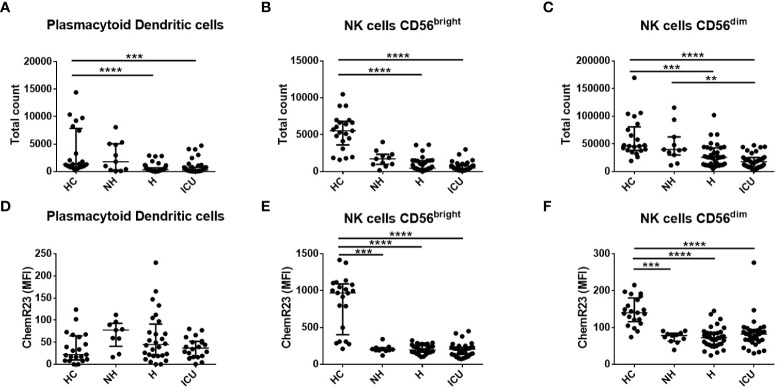
Flow cytometry analysis of the total number of plasmacytoid dendritic cells (pDC), natural killer (NK) cells CD56^bright^ and NK cells CD56^dim^ and their expression of ChemR23 in COVID-19 patients and healthy controls (HC) at day 1. **(A–C)**. Measurements by flow cytometry of total counts of pDCs, NK cells CD56^bright^ and CD56^dim^. **(D–F)**. The expression of ChemR23 was evaluated by mean fluorescence intensity (MFI). Data are presented as median with interquartile range and statistics analysis was performed using Kruskal-Wallis test followed by Dunn’s *post-hoc* test. Non-hospitalized (NH), n=11; hospitalized non-intensive care unit (H), n=36; hospitalized in intensive care unit (ICU), n=35 and healthy controls (HC), n=21. **: p<0.01; ***: p<0.001; ****: p<0.0001.

A deeper analysis of different leukocyte populations using flow cytometry analysis at D1 indicated that ICU patients had higher levels of classical (CD14^+^CD16^-^) (p<0.01) and non-classical (CD14^-^CD16^+^) monocytes (p<0.0001) (**Supplemental Figures 1A, E**) as compared to HC. On the contrary, H and ICU patients had lower levels of pDCs (CD103^+^) (p<0.0001) and both subtypes of NK cells (NK CD56^bright^ and NK CD56^dim^) (p<0.0001) than HC (**Figures 1A–C**). ICU patients had also lower levels of mDCs type 2 (CD11c^+^CD141^-^) as compared to HC (**Supplemental Figure 1I**). A small decrease in the number of intermediate monocytes (CD14^+^CD16^+^) was observed in the H group compared to HC (p<0.05), while no significant differences were observed for mDCs type 1 (CD11c^+^CD141^+^, **Supplemental Figure 1C, G**).

These immune cell populations were also evaluated from BALs from 9 controls and 19 COVID-19 patients. The main clinical characteristics and biological parameters from these subjects are detailed in **Supplemental Table 3**. We observed a higher proportion of PMN (31.0 (22.8-49.4) versus 5.7 (2.6-10.2) %, median (95% CI), p<0.0001) and a lower proportion of macrophages (32.0 (14.0-62.0) versus 80.0 (68.0-86.0) %, median (95% CI), p<0.001) in BAL from COVID-19 patients compared to HC (**Supplemental Figures 2A, B**). No significant difference on percentages of the different subtypes of DCs and NK cells was observed, except for mDC type 2 that were lower in COVID-19 patients (p<0.05) (**Supplemental Figures 2C, E, G, I, K**). Moreover, no significant difference in the expression of ChemR23 was observed between COVID-19 and HC in the cell types analyzed.

### Chemerin expression is increased in plasma from COVID-19 patients and is correlated with inflammation and disease severity

**Figure 2:**
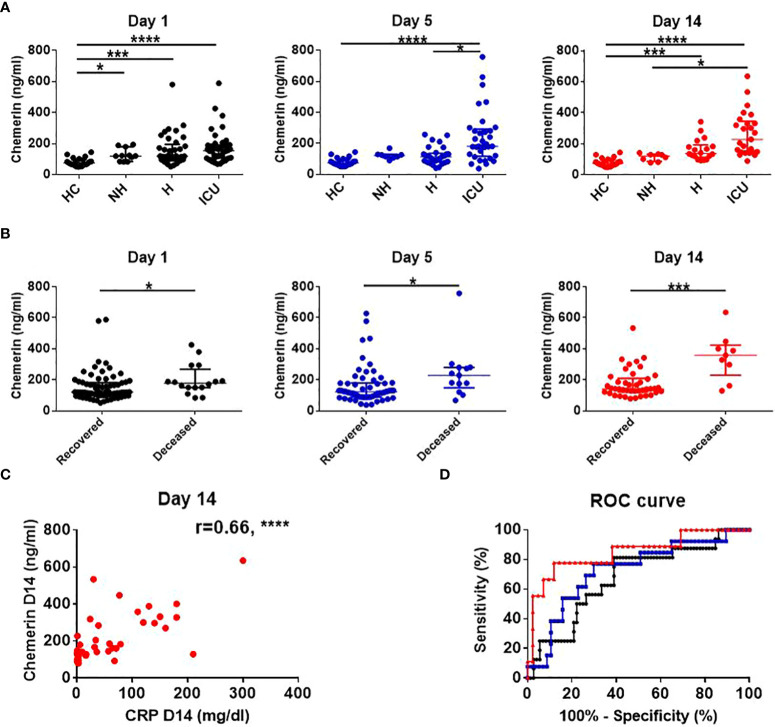
Chemerin assessment and its association with risk of death and inflammation. **(A)** Time course assessment of chemerin concentration in plasma of healthy controls and subgroups of COVID-19 patients. Of note, data from HC were obtained at only one time point. Data are presented as median with interquartile range and statistical analysis was performed using Kruskal-Wallis test followed by Dunn’s *post-hoc* test. **(B)** Comparison of plasma chemerin levels in recovered versus deceased COVID-19 patients. Data are presented as median with interquartile range and statistics analysis was performed using Mann-Whitney test. **(C)** Prediction of mortality by chemerin concentration at day 1 (black), day 5 (blue) and day 14 (red) using a ROC analysis **(D)** Correlation between chemerin concentrations and inflammation, estimated by C-reactive protein (CRP) concentration at day 14. The r corresponds to the Spearman coefficient for non-parametric correlation. Non-hospitalized (NH), n=11; hospitalized non-intensive care unit (H), n=37; hospitalized in intensive care unit (ICU), n=40; healthy controls (HC), n=21. *: p<0.05; ***: p<0.001; ****: p<0.0001.

At all the time points analyzed, plasma chemerin levels were higher and significantly different in COVID-19 patients compared to HC (COVID-19 D1: 125.1 ng/mL (118.4-161.1); COVID-19 D5: 130.3 ng/mL (115.7-175.4); COVID-19 D14: 149.4 ng/mL (135.5-186.4) versus HC: 75.9 ng/mL (63.7-94.3), median of all patients (95% CI), p<0.0001). When comparing each subgroup of COVID-19 patients with HC, plasma chemerin levels were increased in the ICU group at all timepoints (D1, D5 and D14), in the H group at D1 and D14 and in the NH group at D1 (**Figure 2A** and **Supplemental Table 4**). A trend for higher chemerin’s values in ICU patients compared to H and NH patients was also present with a significant difference at D5 between H and ICU patients (p<0.01) and at D14 between NH and ICU patients (p<0.05) (**Figure 2A** and **Supplemental Table 4**). Regarding chemerin concentrations in all COVID-19 patients, they were significantly higher at D14 as compared to D1 (p<0.05) (**Supplemental Figure 3A**). When analyzing within each subgroup of patients, an increase in chemerin concentration was observed in H patients between D1 and D14 and between D5 and D14 (p<0.05). A non-significant trend was observed for ICU patients between D5 and D14 (**Supplemental Figures 3B–D**).

Since higher chemerin levels are associated with metabolic syndrome (27), we investigated the existence of such possible associations in our population. Hypertension was found in 45/109 patients (41.3%), predominantly in H and ICU groups (p<0.0001) and chemerin levels were higher in patients with hypertension (Hypertension: 164.7 ng/mL (141.3-183.5) versus non-hypertension: 118.4 ng/mL (110.8-127.0), median (95% CI), p<0.0001). 34/109 (31.2%) patients had diabetes, mostly belonging to H and ICU groups but no difference in chemerin levels was observed. Other comorbidities were uncommon: CKD in 8/109 (7.3%), COPD in 6/109 (5.5%) and immunosuppression in 10/109 (9.2%) and none was associated with higher chemerin levels. Of note, immunosuppression in our cohort referred to patients with an immunosuppressive treatment (9/109) or having a neoplasia (1/109). In addition, none of our patients presented a hepatic disease such as viral or auto-immune hepatitis, nor cirrhosis.

During the study period, dexamethasone treatment in patients requiring oxygen therapy was introduced to reduce mortality (28). As expected, no NH patient received dexamethasone whereas 20/37 H and 35/40 ICU patients benefited from this treatment. However, this treatment did not modify chemerin levels (147.7 ng/mL (125.6-167.7) versus 158.0 ng/mL (127.2-189.0) in dexamethasone treated and untreated groups respectively, median (95% CI), p=NS).

**Figure 3:**
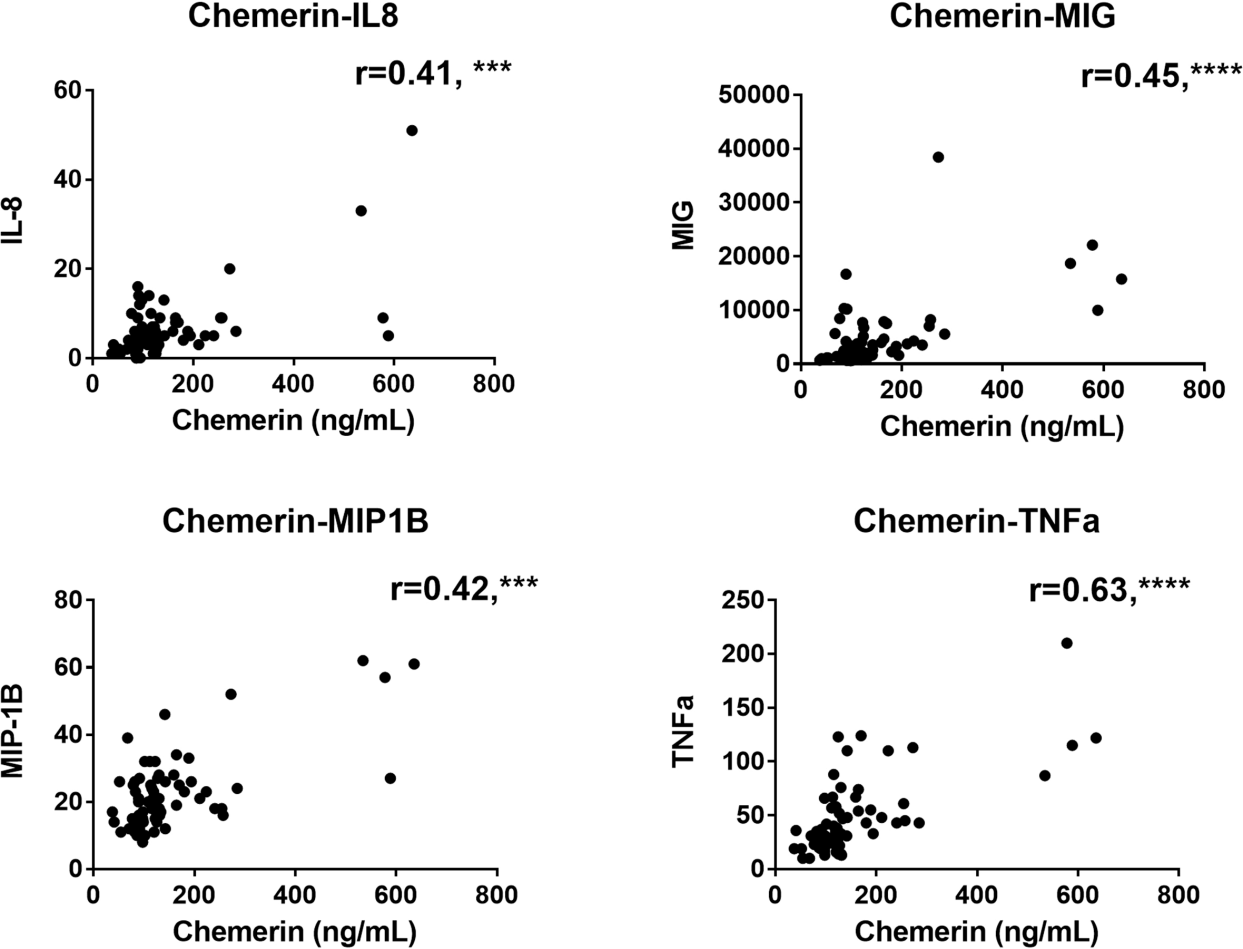
Correlations analysis between chemerin concentration and the levels of different cytokines and chemokines measured by multiplex in plasma of COVID-19 patients at all time-points. The r corresponds to the Spearman coefficient for non-parametric correlation. IL-8: interleukin 8; MIG: monokine induced by interferon-γ; MIP-1β: macrophage inflammatory protein-1β; TNFα: tumor necrosis factor α. N=70 for all analysis. ***: p<0.001; ****: p<0.0001.

We also evaluated the correlation between plasma chemerin concentrations and the levels of various inflammatory markers. Notably, plasma chemerin was strongly correlated with CRP concentration at D14 (r=0.66, p<0.0001) and at all time points, for each increase of 1 mg/dL of CRP, chemerin increased of 209.2 pg/mL (39.4-378.9, median (95% CI), p<0.05) (**Figure 2C**). Levels of chemerin were also strongly and positively correlated with TNFα (r=0.63, p<0.0001) and a moderate positive correlation was found with IL-8 (r=0.41, p<0.001), MIG (r=0.45, p<0.0001) and MIP-1β (CCL4) (r=0.42, p<0.001) (**Figure 3**). Low correlation between chemerin levels and IFN-γ (r=0.32, p<0.01), IL-6 (r=0.37, p<0.01) and MCP-1 (CCL2) (r=0.39, p<0.001) was observed. However, no correlation was shown for IFN-α (r=0.21, p=NS), IL-1β (r=0.25, p=NS), IL-17α (r=0.20, p=NS), IL-10 (r=-0.12, p=NS) and IL-7 (r=0.17, p=NS).

As expected, the duration of hospitalization was lower in the H group compared to the ICU group (6–10) days vs 21.5 (16–28) respectively, median (95% CI), p<0.0001). An ARDS occurred in 38 out of 88 (43.2%) COVID-19 patients, all belonging to the ICU group. Chemerin levels were higher in patients with ARDS than without ARDS at D5 (181,8 (141.3-272.5) versus 115.7 (95.4-129.9) ng/ml, median (95% CI), p<0.001) and D14 (270.8 (162.3-332.2) versus 132.2 (119.8-149.4) ng/ml, median (95% CI), p<0.0001).

Regarding mortality, 16 out of 88 (18%) COVID-19 patients died, 1 from the H group (2.7%) and 15 from the ICU group (37.5%). Chemerin levels were higher in patients who deceased at all time points (**Figure 2B**). Moreover, a univariate logistic regression showed that for each increase of 50 ng/mL of chemerin at D14, the odds of dying increased by 1.78 (1.22-2.61, p<0.01). Age was also associated with the odds of dying, with an increase of 1.06 (1.01-1.10, p<0.05) per added year of age. However, hypertension, diabetes, gender and obesity (BMI ≥ 30kg/m²) were not related to the risk of death (**Table 3**). The final multivariable model was adjusted for age and diabetes, based on the Akaike Information Criterion, and showed that any increase of 50ng/mL of chemerin at D14 was associated with an odd of dying of 2.28 (1.24-4.20, p<0.01) (**Table 3**).

**Table 3:** Risk of death: Univariate logistic regression and multivariate model.

Univariate logistic regression	Odds Ratio (IC95%)	p-value
Chemerin D1 (50ng/mL increase)	1.23 (0.96-1.57)	0.090
Chemerin D5 (50ng/mL increase)	1.20 (0.98-1.46)	0.063
Chemerin D14 (50ng/mL increase)	1.78 (1.22-2.61)	**0.003**
Age	1.06 (1.01-1.10)	**0.019**
Diabetes	1.76 (0.59-5.27)	0.300
Hypertension	1.76 (0.57-5.36)	0.310
Gender	0.81 (0.23-2.79)	0.730
Obesity	1.73 (0.48-6.23)	0.391
**Final Multivariate Model**	**Odds Ratio (IC95%)**	**p-value**
Chemerin D14 (50ng/mL increase)	2.28 (1.24-4.20)	**0.008**
Age	1.08 (0.98-1.18)	0.090
Diabetes	21.74 (0.99-475.90)	0.050

Values in bold indicate statistical significance.

Receiver operating characteristic (ROC) curves were also generated to evaluate the predictive value of plasma chemerin on mortality. This showed that day 14 chemerin’s concentration best estimated mortality risk (AUC=0.85, p<0.01) compared to D1 (AUC=0.68, p<0.05) and D5 concentrations (AUC=0.73, p<0.05) (**Figure 2D**). Thus, a chemerin concentration above 291.4 ng/mL at D14, predicted the risk of death with a sensitivity of 77.78%, a specificity of 88.10%, a positive predictive value of 63.64% and a negative predictive value of 95.00%.

In addition to plasma chemerin analysis, BALs from 9 controls and 19 COVID-19 patients (**Supplemental Table 3**) were used for chemerin assessment. The levels of chemerin on BAL were at least 1000x lower than observed in blood and no statistical difference was observed between chemerin concentrations in the BAL of controls and COVID-19 patients (42 (35–120) versus 44 (35–112) pg/mL respectively, median (95% CI), p=NS).

In order to assess the relation between chemerin and epithelial cells injury, we measured levels of the Krebs Von den Lungen-6 (KL-6) protein, a molecule that is predominantly expressed by damaged alveolar type II cells (29). This analysis was performed on plasma from COVID-19 patients at D1 and D14. KL-6 levels were significantly higher in ICU patients than NH patients at D1 (p<0.05) but no significant difference was shown at D14, even if a trend was emerging (**Supplemental Table 4**). Within ICU patients, there was no significant difference in KL-6 levels at D1 versus D14 (p=NS). Moreover, no correlation between chemerin and KL-6 levels was found (r=0.07, p=NS)

### ChemR23 expression in circulating immune cells from COVID-19 patients

Since we observed higher levels of chemerin in the blood of COVID-19 patients we determined the expression of its main receptor ChemR23 at the surface of immune cell populations of patients and HC. Small increases in ChemR23 expression were found on the classical and intermediate monocyte and mDC populations when comparing some categories of COVID-19 patients and HC (**Supplemental Figures 1B, D, H, J**). No significant differences were found in non-classical monocytes and pDCs (**Figure 1D** and **Supplemental Figure 1F**). Interestingly, ChemR23 expression on NK cells was lower and significantly different on NH, H and ICU patients compared to HC (**Figures 1E, F**). Of note, as previously reported by our group (9, 30), no expression of ChemR23 was reported on PMNs nor lymphocytes.

### Expression of chemerin and ChemR23 in lungs from autopsied COVID-19 patients

Finally, we determined the expression of chemerin and ChemR23 in lung tissues from autopsied COVID-19 patients. Given the important heterogeneity of lung lesions, we selected areas with typical diffuse alveolar damage (DAD) or pneumonia/bronchopneumonia. Areas with DAD were either in exudative phase with hyaline membranes, interstitial and intra-alveolar edema, microthrombi or in organizing phase with interstitial or intra-alveolar proliferation of myofibroblasts and proliferation of type II pneumocytes (26). Lysis phenomena were also observed with essentially a desquamation of epithelial and endothelial cells (ECs).

**Figure 4:**
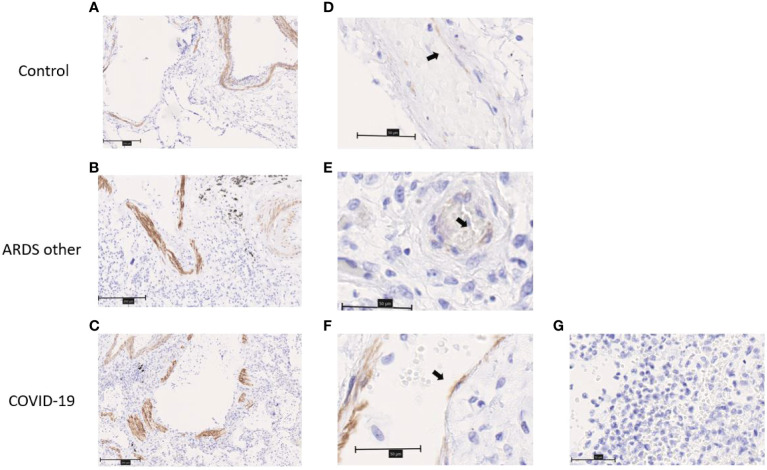
Immunohistochemistry staining of ChemR23 on lung slides from autopsied COVID-19 patients and controls (autopsied patient without major pulmonary lesion (control) and patient with ARDS from another origin (ARDS other)). **(A–C)**. Representative staining of smooth muscle cells from arteries and bronchia. Field magnification 100x. Scale bar: 100µm. **(D–F)**. Representative staining of endothelial cells (black arrows). Field magnification 400x. Scale bar: 50µm. **(G)**. Representative image from area of acute pneumonia in COVID-19 patients, without ChemR23 staining. Field magnification 400x. Scale bar: 50µm.

**Figure 5:**
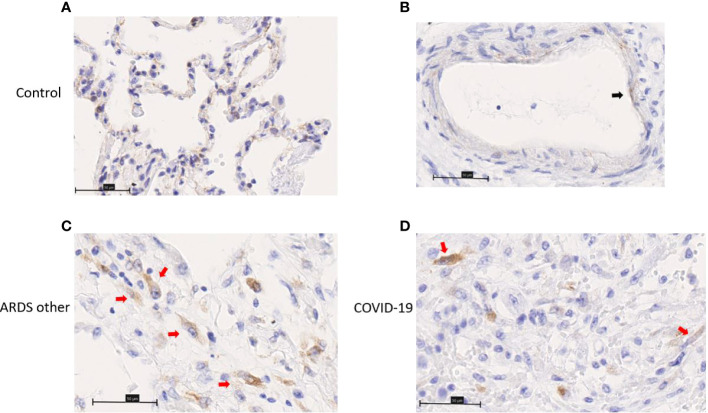
Immunohistochemistry staining of chemerin on lung slides from autopsied COVID-19 patient, patient with ARDS from another origin (ARDS other) and control. **(A)** Representative image of alveolar lining staining. **(B)** Representative image of endothelial cell (black arrow) staining. **(C, D)** Representative image of chemerin staining of spindle cell (red arrows) in organizing phase of diffuse alveolar damage. Field magnification 400x. Scale bar: 50µm.

ChemR23 expression was found on smooth muscle cells of vessels and bronchi (**Figure 4A–C**) and focally on ECs (**Figure 4D–F**). There was no staining observed in the immune cell populations visualized (**Figure 4G**). This staining was similar between healthy lungs, lungs with DAD secondary to COVID-19 or other causes. Chemerin expression was detected on alveolar epithelium of healthy lung controls and focally on EC (**Figures 5A, B**). However, no clear staining was observed on alveolar epithelium and EC from COVID-19 patients. Interestingly, in slides from COVID-19 and other ARDS patients, spindle cells (fibroblasts or myofibroblasts) in the organizing phase of DAD strongly expressed chemerin (**Figures 5C, D**). Given that no fibrosis was present in control lungs, no spindle cells were observed.

## Discussion

In the present study we analyzed the chemerin/ChemR23 system in a cohort of confirmed COVID-19 patients. We presently showed, for the first time, that chemerin concentrations are elevated in plasma of COVID-19 patients and are associated with the severity of the disease, inflammation and are an independent risk factor of mortality.

Our observation of elevated levels of chemerin in COVID-19 patients are in contradiction with a recent publication, where serum chemerin levels were lower in COVID-19 patients at day 1 of hospitalization than in healthy controls (31). However, chemerin values in the healthy controls in this study were superior to those described in the literature. Thus, while the described values are usually between 60.0 and 130.0 ng/mL (10, 12, 32–34), which correspond to our results (75.9 ng/mL (63.7-94.3)), in the study of Kukla et al., control values for chemerin were around 373.0 ng/mL. Moreover, we determined chemerin concentration in plasma, whereas Kukla et al. measured it on serum. Chemerin can be degraded by many proteases that are possibly increased in acute inflammatory state. If samples are not handled properly, this could explain why chemerin concentrations were lower in their COVID-19 patients compared to their controls. Further studies evaluating the dosage of chemerin in plasma versus serum in patients with an acute inflammatory state are needed to address this hypothesis. Finally, discrepancy among data could also results from difference in subgroup size (40 patients in our ICU group vs 9 in Kukla et al.) and the fact that possibly more severe patients could be included in our study taken into consideration that we are part of a tertiary care center.

Increased concentration of chemerin were already observed in many other inflammatory conditions and the levels observed in the COVID-19 patients of our cohort (ranging from 125.1 to 149.4 ng/mL) are in line with those observed in rheumatoid arthritis (154 ng/mL (35)), psoriasis (125.3-196 ng/mL) (32, 33, 36), Crohn’s disease (140 ng/mL) (12), ulcerative colitis (124 ng/mL) (12), type 2 diabetes (144 ng/mL) (37) and acute myocardial infarction (173.8 ng/mL) (34). As previously outlined, chemerin levels were correlated with markers of inflammation as CRP but also pro-inflammatory cytokines as TNF-α (27, 37, 38). Moreover, chemerin levels tended to increase with the severity of the disease (e.g. admission to ICU and development of an ARDS) and were higher in deceased patients as compared to subjects that recovered from the disease. Interestingly, in COVID-19 patients, chemerin levels increased significantly with time but this increase was only significant for H patients and not in ICU patients, even is a trend is seen between D5 and D14. Our hypothesis is that the higher mortality rate observed in ICU group (18%) compared to H group (2.7%) led to a loss of late samples that could have shown higher chemerin levels (ICU D1 n=40; ICU D5 n=35; ICU D14 n=25). The decrease in the number of samples in D14 also led to a lower statistical power.

Importantly, chemerin concentrations at day 14 was associated with risk of mortality in both univariate logistic regression and multivariate analysis. Moreover, we identified a chemerin threshold of 291.4 ng/mL at day 23 after initial symptoms that could predict mortality with a high sensitivity, high specificity and an interesting high negative predictive value. In our cohort, patients with hypertension had higher chemerin levels. This is in agreement with a previously study demonstrating that chemerin was independently associated with hypertension (10).

The increased levels of chemerin detected in plasma of COVID-19 patients were not observed in BAL and they were much lower than the ones observed in blood. This could be explained by the important dilution factor of the BAL and possible degradation of chemerin by proteases. Also, no BAL were obtained from healthy subjects and the number of BAL collected was relatively small and from heterogenous patients (some having an added bacterial infection, or variable duration of hospitalization). All of these could interfere with our results and further studies are therefore necessary to determine chemerin levels in the BAL of these patients. Of note, we were unable to find studies where chemerin levels were measured in BAL for comparison.

We previously showed that ChemR23 KO mice exhibited a similar immune signature as severe forms of COVID-19 with development of a cytokine storm and lower levels of pDCs and lymphocytes (14). We confirmed this immune signature in our cohort of COVID-19 patients. Particularly, regarding the total number of immune cell populations, we found lower levels of pDCs and NK cells from COVID-19 patients as previously described in the literature (39, 40). Our results are also consistent with a recent study, using partly the same cohort of patients (23). Additionally, we reported lower expression of ChemR23 on NK cells from COVID-19 patients. This could be the result of an internalization of the receptor associated to the presence of higher chemerin concentrations in COVID-19 patients. However, lower ChemR23 expression was not observed on other immune cell types. Another hypothesis could be the migration of NK cells with a high expression of ChemR23 to inflamed tissues as the lung. Unfortunately, we could not confirm this hypothesis through BAL analysis, as small cell populations such as pDCs or NK cells may have been masked by the dominance of neutrophils and monocytes/macrophages, as previously described in literature (41). Finally, ChemR23 decrease on NK cells could be related to a modulating effect of pro-inflammatory cytokines or chemokines. However, there is no available information regarding the regulation of the expression of ChemR23 on NK cells by other molecules than chemerin.

Our histological analysis confirmed the expression of ChemR23 in lung endothelial cells (EC) of both HC and COVID-19 patients. Interestingly, endothelial activation and dysfunction are involved in the physiopathology of severe viral pneumonia and seems to be more prominent in cases of severe SARS-CoV-2 infection. Indeed, it has been demonstrated that patients who died from COVID-19 had predominant angiocentric inflammation and more severe endothelial injury than patients with influenza pneumonia of equal severity (42). It has been previously described that the chemerin/ChemR23 system mediates anti-inflammatory effects on ECs *via* activation of the Akt/eNOS/NO pathway (7). Additionally, recent results from our group in the oncology field showed that chemerin displays anti-tumoral properties that are also mediated by ECs (43, 44). Therefore, the role of the chemerin/ChemR23 on endothelial dysfunction observed in the COVID-19 patients deserves further studies.

Besides ECs we observed that chemerin is expressed in fibroblasts/myofibroblasts in lesions of late DAD and ChemR23 is also expressed on smooth muscle cells. Recent studies demonstrated that epithelial-to-mesenchymal and endothelial-to-mesenchymal transition were present in patients with severe COVID-19 (45, 46) and that these patients are more likely to evolve to pulmonary fibrosis (47). The role of the chemerin/ChemR23 system in the physiopathology of lung fibrosis was not yet studied and overall, the role of this system on fibrosis is not clear. Few studies on liver-related diseases correlates the expression levels of chemerin or some of its receptors either positively or negatively with the development of liver fibrosis (48–50). One study correlates kidney fibrosis with increased levels of chemerin/ChemR23 in rat models (51), while in a mouse model of wound healing administration of the active form of chemerin promotes skin repair and reduce scarring (52). Therefore, it would be of interest to verify if the chemerin/ChemR23 system could be involved in lung fibrosis following ARDS. Of note, we did not detect any staining of ChemR23 on immune cells on our histological analysis. This can be explained by the fact that the vast majority of immune cells observed were PMN and although there is a controversy in the literature regarding this issue, in our experiments no expression of ChemR23 on PMNs was observed (9, 30).

Our study has few limitations. It is a monocentric study with a relatively small number of patients, particularly in the HC and NH groups, that may decrease the robustness of our results. Nevertheless, our patient cohort is quite representative of hospitalized COVID-19 patients with a predominance of elderly men with numerous comorbidities such as hypertension and diabetes (16). As well, our HC group differs significantly from COVID-19 patients, both for age and the presence of comorbidities. However, analysis of the intrinsic mortality of each comorbidity (age, gender, hypertension and diabetes), only aging led to a significant increase in mortality. Importantly, patients with chemerin levels at D14 higher than 291.4 ng/mL had the highest percentage of mortality (**Supplemental Figure 4**). This was confirmed by univariate logistic regression and the multivariate model. We selected patients from first and second wave of SARS-CoV-2 infection and admission criteria to ICU and treatments changed during this period. Although dexamethasone treatment did not modify chemerin levels, other factors could be involved and bias our results. Unfortunately, flow cytometry analysis of BAL was limited due to the low number of cells in these samples and no BAL samples were obtained from healthy participants. Finally, regarding lung samples used for IHC, to avoid infection of the staff, autopsies of COVID-19 patients were performed 72 hours after the patient’s death. To avoid biases in the analysis, we therefore selected control tissues coming from late autopsies. However, due to the late treatment of the samples, a desquamation of epithelial and/or endothelial cells was observed in tissues from COVID-19 patients and controls, probably affecting the quality of the staining.

In conclusion, our study demonstrates that increased plasma chemerin levels are a marker of severity and death in COVID-19 patients. However, multicentric studies and validation cohorts are needed to affirm chemerin as a biomarker of severity and death to be used in daily clinical practice. Further studies are also needed to identify the precise mechanisms by which the chemerin/ChemR23 system affects ARDS secondary to viral pneumonia and its possible role in lung fibrosis.

## Data availability statement

The original contributions presented in the study are included in the article/**Supplementary Material**. Further inquiries can be directed to the corresponding author.

## Ethics statement

The studies involving human participants were reviewed and approved by Local Ethical Committee, (P2020/238 and P2020/232), Erasme Hospital. The patients/participants provided their written informed consent to participate in this study.

## Author contributions

The protocol was designed by PL, VC, MP, AK, and BB. The clinical data collection was performed by PL, SM, and CO. Technical support and material were provided by VC, VO, ND, VDM, AM, CD, FM, PB, and IS. Experiments were realized by PL, NA and NDV. Data analysis was performed by PL, NA, VC, NDV, MR, MP, AK, and BB. The manuscript was written by PL, AK, and BB. All authors critically reviewed the manuscript and approved its final version.

## Funding

PL has a PhD scholarship from F.N.R.S. (Belgian National Fund for Scientific Research) and is a grant holder of Fonds Erasme. NA was supported partially by a ULB grant and partially from Fonds Erasme. VC was supported by a COVID-19 ULB grant. ND is a post-doctoral clinical master specialist of the F.N.R.S. V.d.M. received a grant from the Foundation Jaumotte-Demoulin. CD is a Senior Research Associate with the F.N.R.S. The CMMI is supported by the European Regional Development Fund and the Walloon Region (Wallonia-biomed, #411132-957270, project “CMMI- ULB”). The department of Pathology of Erasme hospital (PL, IS, and MR) was supported by Fonds Yvonne Boël, Foundation ULB and Fonds Erasme. BB and FM received grants from Foundation ULB and Fonds Erasme. NDV obtained a LHUB-ULB research grant. BB received financial support from Amgen and Boehringer Ingelheim. This article was published with the financial support of the Fonds Erasme.

## Acknowledgments

We would like to thank Audrey Godefroid, Anne Van Praet and Noémie Law-Weng-Sam (LoVMI) for blood samples handling, biobanking and Multiplex analysis; Flavienne Sandras (Department of Pathology, Erasme Hospital) for bronchoalveolar lavages and tissues biobanking; Justine Allard and Amandine Collin (DIAPath, CMMI) for technical assistance on immunohistochemistry; Mélina Houinsou Hans (Department of Biomedical Research, Erasme Hospital) for help with statistical analysis.

## Conflict of interest

BB received a financial support from Amgen and Boehringer Ingelheim. These funders were not involved in the study design, collection, analysis, interpretation of data, the writing of this article and the decision to submit for publication.

The remaining authors declare that the research was conducted in the absence of any commercial or financial relationships that could be construed as a potential conflict of interest.

## Publisher’s note

All claims expressed in this article are solely those of the authors and do not necessarily represent those of their affiliated organizations, or those of the publisher, the editors and the reviewers. Any product that may be evaluated in this article, or claim that may be made by its manufacturer, is not guaranteed or endorsed by the publisher.
