# Supplementary material for: Chemerin plasma levels are increased in COVID-19 patients and are an independent risk factor of mortality
Source: Front Immunol. 2022 Aug 12;13:941663. doi: 10.3389/fimmu.2022.941663 (PMC9412239; doi:10.3389/fimmu.2022.941663)
Supplement: Supplementary file 1 [file DataSheet_1.docx]

| **Supplemental Table 1. Baseline characteristics and comorbidities with multiple comparisons between groups.** | | | | | | | | | | | |  |
| --- | --- | --- | --- | --- | --- | --- | --- | --- | --- | --- | --- | --- |
| **Baseline characteristics and comorbidities** | **Healthy controls (n=21)** | **Non-hospitalized COVID-19 patients (n=11)** | **Hospitalized non-ICU COVID-19 patients (n=37)** | **ICU COVID-19 patients (n=40)** | **p-value all** | **p-value HC versus NH** | **p-value HC versus H** | **p-value HC versus ICU** | **p-value NH versus H** | **p-value NH versus ICU** | **p-value H versus ICU** |  |
|  |  |  |  |  |  |  |  |  |  |  |  |  |
| Age (years)° | 38.6 ± 7.9 | 42.2 ± 10.9 | 57.6 ± 14.8 | 60.5 ± 10.9 | **<0.001** | 0.488 | **<0.001** | **<0.001** | **<0.001** | **<0.001** | 0.488 |  |
| Gender (M/F) | 8/3 | 8/3 | 24/13 | 31/9 | **<0.001** | **-** | **-** | **-** | - | - | - |  |
| BMI (kg/m²)* | - | 28.4 (22.4-33.9) | 27.7 (26.0-30.8) | 29.5 (27.6-33.1) | 0.117 | - | - | - | 1.000 | 0.355 | 0.203 |  |
| Smoking n (%) | - | 2 (18) | 4 (12) | 8 (25) | 0.379 | - | - | - | - | - | - |  |
| Hypertension n (%) | 0 (0) | 2 (18) | 19 (51) | 24 (60) | **<0.001** | - | - | - | - | - | - |  |
| Diabetes n (%) | 0 (0) | 1 (9) | 10 (27) | 23 (57.5) | **<0.0001** | - | - | - | - | - | - |  |
| Immunosuppression n (%) | 0 (0) | 0 (0) | 5 (13.5) | 5 (12.5) | 0.088 | - | - | - | - | - | - |  |
| COPD n (%) | 0 (0) | 0 (0) | 4 (11) | 2 (5) | 0.303 | - | - | - | - | - | - |  |
| CKD n (%) | 0 (0) | 0 (0) | 6 (16) | 2 (5) | 0.327 | - | - | - | - | - | - |  |
|  |  |  |  |  |  |  |  |  |  |  |  |  |
| °Parametric data presented as mean ± Standard deviation (SD). ^$^Non-parametric data presented as median with confidence interval of 95%. BMI: body mass index; CKD: chronic kidney disease; COPD: chronic obstructive pulmonary disease. Statistics analysis was performed using one-way ANOVA followed by Tukey’s post-hoc test or Kruskal-Wallis followed by Dunn’s post-hoc test, according to the distribution of data, for non-categorical variables. Chi-square test was applied for categorical variables. | | | | | | | | | | | | |
|  | | | | | | |  |  |  |  |  |  |

| **Supplemental Table 2. Chemerin and main biological parameters at admission with multiple comparisons between groups.** | | | | | | | |
| --- | --- | --- | --- | --- | --- | --- | --- |
|  | **Non-hospitalized COVID-19 patients (n=11)** | **Hospitalized non-ICU COVID-19 patients (n=37)** | **ICU COVID-19 patients (n=40)** | **p-value** | **p-value NH versus H** | **p-value NH versus ICU** | **p-value H versus ICU** |
|  |  |  |  |  |  |  |  |
| KL-6 D1 (U/mL)* | 301 (154-422) | 377.5 (254-547) | 577 (422-760) | **0.004** | 0.590 | **0.014** | **0.042** |
| Saturation (%)* | 99 (97-100) | 94 (92-95) | 88 (80-92) | **<0.001** | **0.003** | **<0.001** | **0.009** |
| Hemoglobin (g/dL)° | 13.2 ± 2.8 | 12.8 ± 1.9 | 12.6 ± 2.2 | 0.156 | 0.707 | 0.181 | 0.922 |
| WBC (10³/mm³)* | 5.0 (3.5-9.1) | 5.3 (4.8-6.8) | 11.3 (8.9-13.1) | **<0.001** | 1.000 | **0.002** | **<0.001** |
| PMN (10³/mm³)* | 2.9 (1.7-5.8) | 4.3 (3.1-5.1) | 8.9 (7.5-11.2) | **<0.001** | 0.902 | **<0.001** | **<0.001** |
| Lymphocytes (10³/mm³)* | 1.5 (1.1-2.3) | 0.8 (0.7-1.0) | 0.8 (0.6-0.8) | **<0.001** | **0.001** | **<0.001** | 1.000 |
| Platelets (10³/mm³)* | 194 (115-270) | 190.5 (153-213) | 256 (214-336) | **<0.001** | 1.000 | 0.137 | **<0.001** |
| CRP (mg/L)* | 11.5 (1.9-59) | 69 (38-88) | 135 (97-180) | **<0.001** | **0.039** | **<0.001** | **<0.001** |
| Ferritin (µg/L)* | NA | 604 (386-1261) | 1113 (834-1670) | **0.008** | NA | NA | **0.008** |
| LDH (U/L)* | 214 (153-335) | 324.5 (278-370) | 435 (366-520) | **<0.001** | **0.014** | **<0.001** | **0.004** |
| ALT (U/L)* | 23 [17-33] | 27 [21-30] | 46 [27-59] (39) | **0.014** | 1.000 | **0.050** | 0.053 |
| AST (U/L)* | 24 (17-38) | 34.5 (24-41) | 40 (34-55) | **0.019** | 0.304 | **0.020** | 0.346 |
| GGT (U/L)* | 50.50 (48-53) | 50 (33-67) | 70 (43-105) | 0.115 | 1.000 | 1.000 | 0.120 |
| Total bilirubin (mg/dL)* | 0.6 (0.2-0.9) | 0.4 (0.4-0.5) | 0.6 (0.4-0.7) | 0.548 | 1.000 | 1.000 | 0.831 |
| CK (U/L)* | 188 (53-470) | 91 (59-184) | 129 (79-335) | 0.342 | 0.893 | 1.000 | 0.536 |
| D-dimer (ng/mL)* | NA | 543 (440-1041) | 1182 (963-2528) | **<0.001** | NA | NA | **<0.001** |
| Creatinine (mg/dL)* | 0.9 (0.7-1.2) | 0.8 (0.8-1.0) | 0.9 (0.7-1.0) | 0.793 | 1.000 | 1.000 | 1.000 |
| Urea (mg/dL)* | 24 (18.4-26.9) | 33.4 (28.1-40.5) | 41.7 (33.3-55.3 | **0.002** | 0.129 | **0.002** | 0.134 |
| GFR (mg/dL/1,73m²)* | 93 (69-118) | 93 (82-100) | 84.5 (69-99) | 0.574 | 1.000 | 1.000 | 1.000 |
| Cholesterol total (mg/dL)° | 148.5 ± 55.72 | 158.7 ± 39.17 | 149 ± 39.7 | 0.705 | 0.876 | 0.982 | 0.809 |
| Triglycerids (mg/dL)* | 69 (10.3-162.7) | 130 (117.4-166.2) | 150.5 (131.7-237) | 0.123 | 0.275 | 0.124 | 1.000 |
| °Parametric data presented as mean ± Standard deviation (SD). ^$^Non-parametric data presented as median with confidence interval of 95%. ALT: alanine transaminase; AST: aspartate transaminase; CK: creatinin kinase; CRP: C-reactive protein; GFR: glomerular filtration rate (CKD-epi formula); GGT: gamma-glutamyl transferase; KL-6: Krebs Von den Lungen 6; LDH: lactate dehydrogenase; PMN: polymorphonuclear cell; SpO2: median peripheral oxygen saturation; WBC: white blood cell. Statistics analysis was performed using one-way ANOVA followed by Tukey’s post-hoc test or Kruskal-Wallis followed by Dunn’s post-hoc test, according to the distribution of data. | | | | | | | |

**Supplemental Table 3. Baseline characteristics, comorbidities and biological parameters of BAL patients cohort.**

|  | **Controls (n=9)** | **COVID-19 patients (n=19)** | **p-value** |
| --- | --- | --- | --- |
| Age (years) | 68 (56.5-73.0) | 60.0 (53.0-69.0) | 0.268 |
| Gender (M/F) | 5/4 | 15/4 | 0.371 |
| BMI (kg/m²) | 24.0 (20.0-34.1) | 29.4 (24.0-33.0) | 0.202 |
| Hypertension n (%) | 3 (33) | 13 (68) | 0.114 |
| Diabetes n (%) | 2 (22) | 3 (16) | 1.000 |
| Immunosuppression n (%) | 2 (22) | 7 (37) | 1.000 |
| WBC (10³/mm³) | 8.1 (3.6-9.3) | 10.6 (6.1-15.4) | 0.319 |
| PMN/lymphocytes (10³/mm³) | 2.8 (1.4-10.1) | 9.6 (4.4-23.0) | **0.004** |
| CRP (mg/L) | 1.4 (0.9-21.0) | 200.0 (150.0-230.0) | **0.003** |
| Ferritin (µg/L) | 93.0 (18.0-541.0) | 798.0 (393.0-2622.0) | **0.004** |
| LDH (U/L) | 217.0 (176.0-378.0) | 421.0 (309.0-535.0) | **0.007** |
| CK (U/L) | 58.0 (26.0-103.0) | 53.0 (26.0-281.0) | 0.743 |
| Creatinine (mg/dL) | 0.9 (0.6-1.8) | 0.8 (0.6-1.3) | 0.768 |
| GFR (mg/dL/1,73m²) | 71.0 (53.0-91.0) | 91.0 (53.0-108.0) | 0.745 |
| Cholesterol total (mg/dL) | 152.8 ± 22.7 | 183.6 ± 47.1 | 0.199 |
| Triglycerids (mg/dL) | 88.0 (65.0-207.0) | 156.0 (64.7-346-0) | 0.171 |
| Chemerin (pg/mL) | 44.0 (35.0-112.0) | 42.0 (35.0-120.0) | 0.990 |

Data are presented as median with confidence interval of 95%. BAL: bronchoalveolar lavage; BMI: body mass index; CK: creatinin kinase; CRP: C-reactive protein; GFR: glomerular filtration rate (CKD epi formula); LDH: lactate dehydrogenase; PMN: polymorphonuclear cells; WBC: white blood cells. Statistics analysis was performed using Mann-Whitney test for non-categorical variables and Fisher’s exact test was applied for categorical variables.

| **Supplemental Table 4. Chemerin and KL-6 concentrations in plasma from COVID-19 patients and healthy controls.** | | | | | | | |  |
| --- | --- | --- | --- | --- | --- | --- | --- | --- |
|  | **Non-hospitalized COVID-19 patients (n=11)** | **Hospitalized non-ICU COVID-19 patients (n=37)** | **ICU COVID-19 patients (n=40)** | **p-value all** | **p-value NH versus H** | **p-value NH versus ICU** | **p-value H versus ICU** |  |
|  |  |  |  |  |  |  |  |  |
| Chemerin D1 (ng/mL) | 120.1 (83.3-189) | 118.9 (102.2-161.1) | 154.3 (120.2-185) | 0.320 | 1.000 | 0.693 | 0.630 |  |
| Chemerin D5 (ng/mL) | 120.7 (90.0-169.8) | 102.5 (83.7-131.3) | 181.8 (141.3-265.8) | **0.001** | 1.000 | 0.167 | **0.001** |  |
| Chemerin D14 (ng/mL) | 121.8 (79.1-142.2) | 138.9 (119.8-183.5) | 228.5 (149.4-328) | **<0.001** | 0.306 | **<0.001** | **0.240** |  |
| KL-6 D1 (U/mL) | 301 (154-422) | 377.5 (254-547) | 577 (422-760) | **0.004** | 0.590 | **0.014** | **0.042** |  |
| KL-6 D14 (U/mL) | 363.5 (283-844) | 413 (264-666) | 455.5 (376-827) | 0.404 | 1.000 | 0.823 | 0.839 |  |
| Data presented as median with confidence interval of 95%. KL-6 : Krebs Von den Lungen 6. Statistics analysis was performed using Kruskal-Wallis test followed by Dunn’s post-hoc test. | | | | | | | |  |
|  | | |  |  |  |  |  |  |


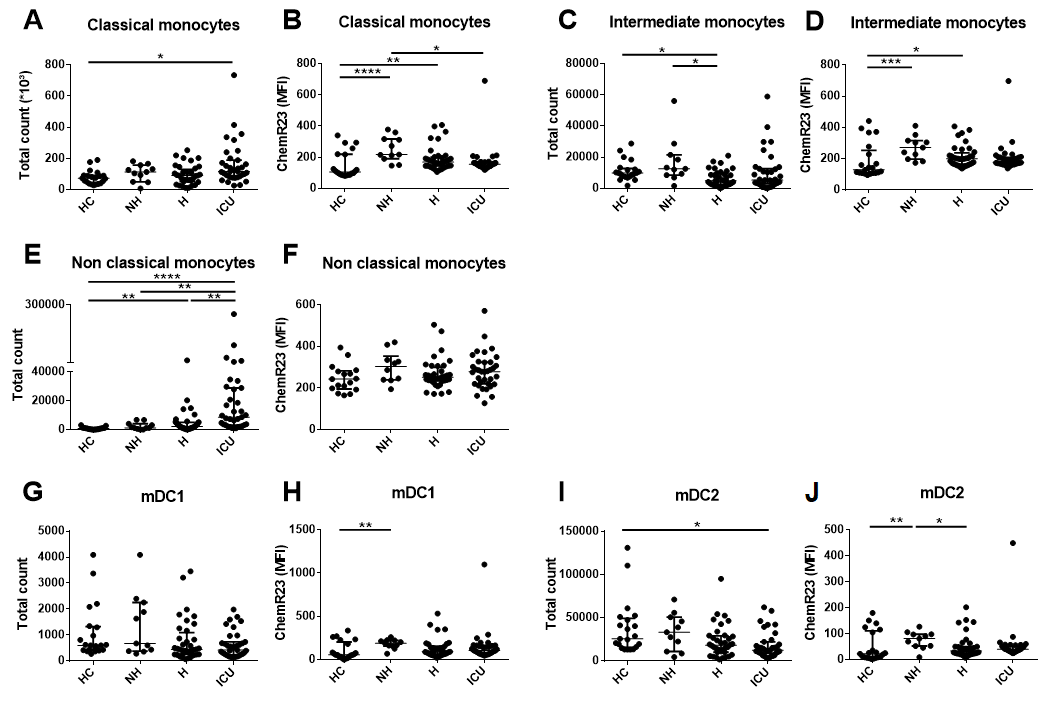


**Supplemental Figure 1.** Flow cytometry analysis of the total number of classical monocytes (CD14^+^), intermediate monocytes (CD14^+^CD16^+^), non classical monocytes (CD16^+^), myeloid dendritic cells type 1 (mDC1) and myeloid dendritic cells type 2 (mDC2) and their expression of ChemR23 in COVID-19 patients and healthy controls (HC) at day 1. **A,C,E,G,I.** Measurements by flow cytometry of total counts of classical monocytes, intermediate monocytes, non-classical monocytes, mDC1 and mDC2. **B,D,F,H,J.** The expression of ChemR23 was evaluated by mean fluorescence intensity (MFI). Data are presented as median with interquartile range and statistics analysis was performed using Kruskal-Wallis test followed by Dunn’s post-hoc test. Non-hospitalized (NH), n=11; hospitalized non-intensive care unit (H), n=36; hospitalized in intensive care unit (ICU), n=35 and healthy controls (HC), n=21. *: p<0.05; **: p<0.01; ***: p<0.001; ****: p<0.0001.


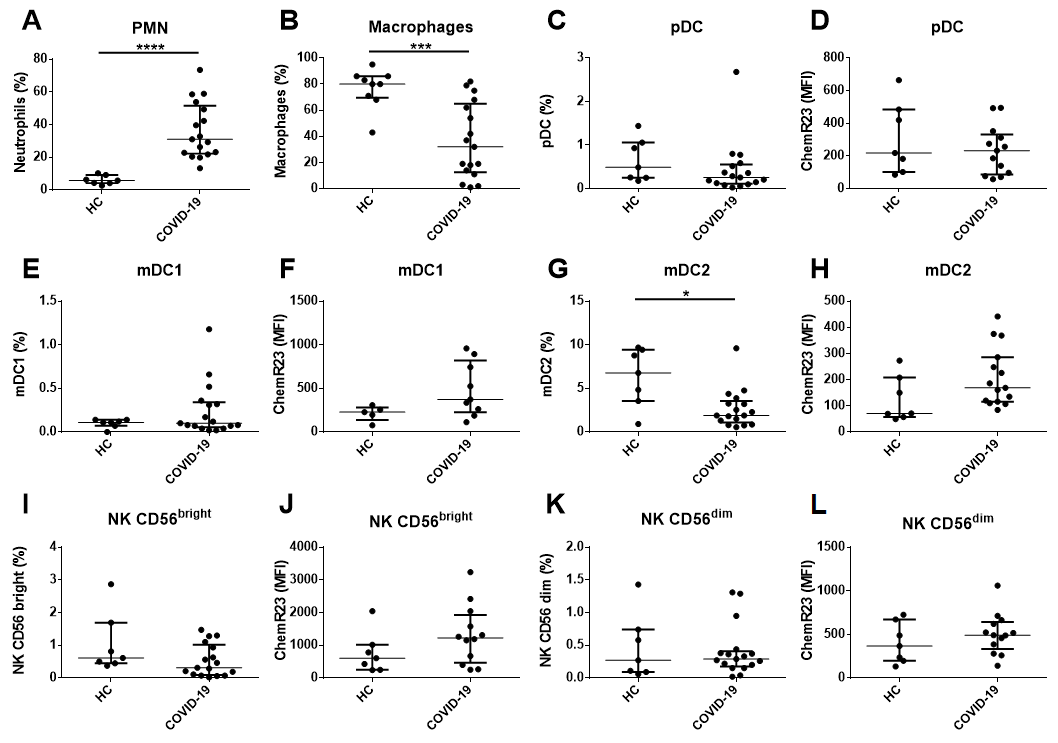


**Supplemental Figure 2.** Analysis of main immune cell populations in bronchoalveolar lavage (BAL) from COVID-19 patients and controls (HC). **A-B.** Routine measurements of polymorphonuclear cells (PMN) and macrophages in BAL, expressed as percentages. **C, E, G, I, K.** Flow cytometry analysis of myeloid dendritic cells type 1 (mDC1), myeloid dendritic cells type 2 (mDC2), plasmacytoid dendritic cells (pDC), natural killer (NK) cells CD56^bright^ and NK cells CD56^dim^ indicated as percentage. **D, F, H, J, L.** The expression of ChemR23 was evaluated by mean fluorescence intensity. Data are presented as median with interquartile range and statistics analysis was performed using Kruskal-Wallis test followed by Dunn’s post-hoc test. Healthy controls (HC), N=7 ; COVID-19 patients, N=17. *: p<0.05; ***: p<0.001; ****: p<0.0001.


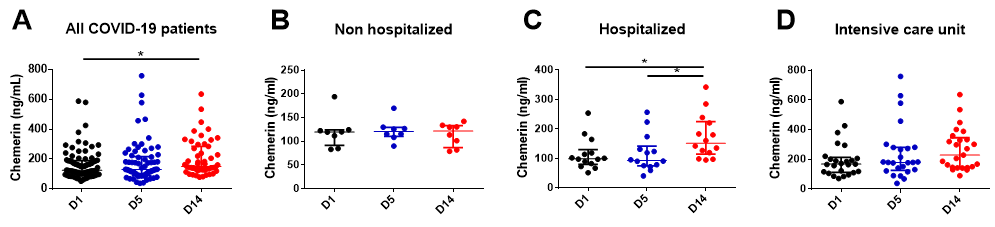


**Supplemental Figure 3.** Analysis of the kinetic of chemerin concentration in plasma from all COVID-19 patients **(A)** and within each subgroup **(B-D)**. Data are presented as median with interquartile range and statistics analysis was performed using Kruskal-Wallis test followed by Dunn’s post-hoc test. *: p<0.05.


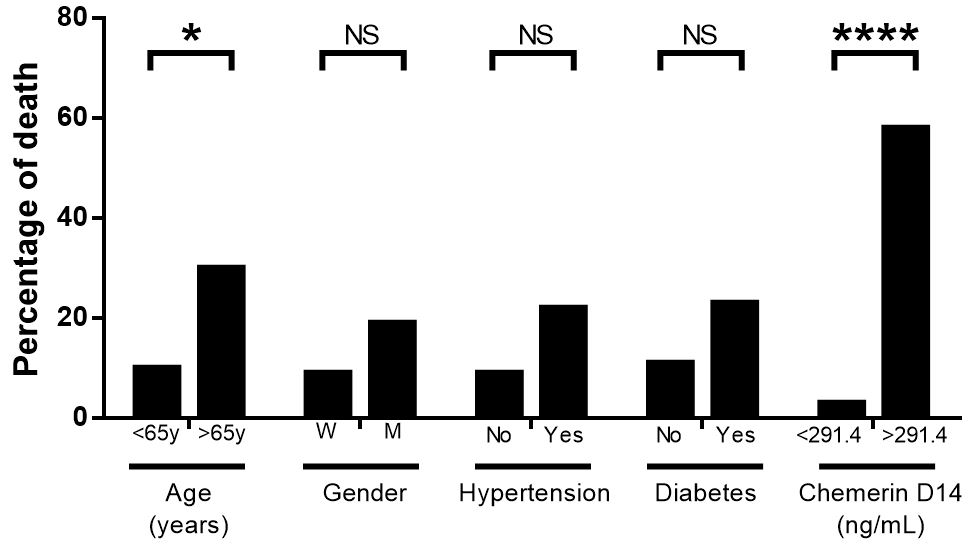


**Supplemental Figure 4.** Death according to comorbidities. Risk of mortality according to age, gender, presence or absence of diabetes or hypertension, or plasma chemerin (over or below the cut-off concentration of 291,4 ng/mL at D14). Statistics analysis was performed using Mann-Whitney test. *: p<0.05; ****: p<0.0001.
